# Flash Communication: Challenging Computational Description of Gold(I)/Gold(III) Catalytic Cycles

**DOI:** 10.1021/acs.organomet.5c00081

**Published:** 2025-06-04

**Authors:** Isabel Arranz, Feliu Maseras, Antonio M. Echavarren

**Affiliations:** † Institute of Chemical Research of Catalonia (ICIQ-CERCA), The Barcelona Institute of Science and Technology, Av. Països Catalans 16, 43007 Tarragona, Spain; ‡ Departament de Química Analítica i Química Orgànica, 202569 Universitat Rovira i Virgili, C/Marcel·lí Domingo s/n, 43007 Tarragona, Spain

## Abstract

We have investigated the mechanism of cross-coupling reactions catalyzed by gold­(I) complexes without the assistance of chelating ligands. Following pioneering studies by Kochi, gold­(I) complexes with simple alkyl phosphines as ligands are considered. The reaction between cinnamyl bromide and PhSnMe_3_ is experimentally shown to take place in the presence of a [Me_3_PAuCl] complex. However, our attempt to characterize computationally, using density functional theory (DFT), a mechanism following a plausible gold­(I)/gold­(III) catalytic cycle unearths an unexpectedly complex situation, showing a large range of energy values computed with different functionals.

## Graphical abstract

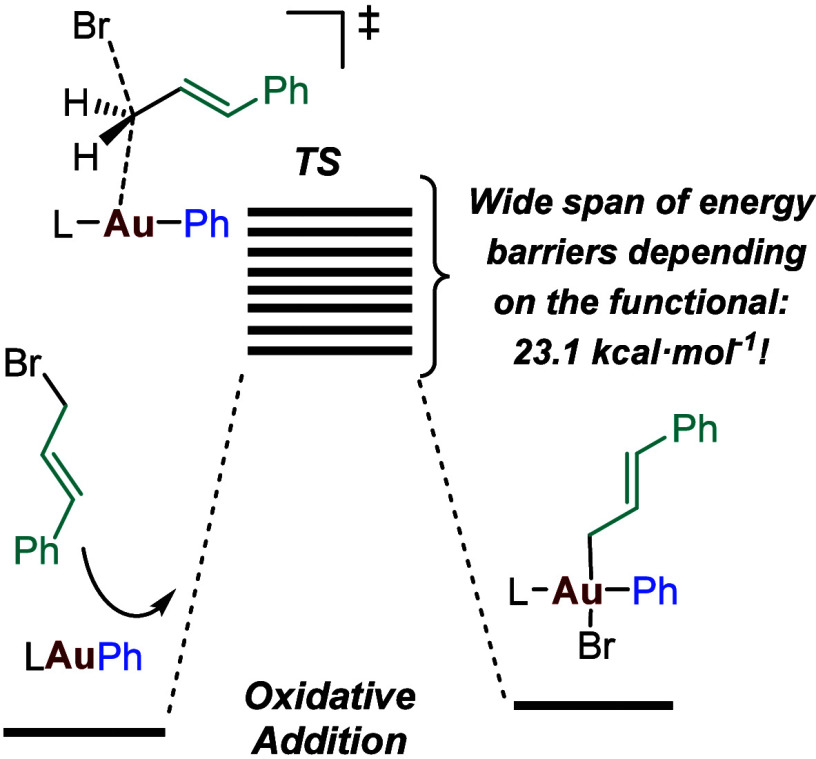

The oxidative addition of organic electrophiles R–X to LAu­(I)­X, where L is a monodentate PR_3_ ligand, a common step in cross coupling with this type of d^10^ complexes, was computed to require high activation barriers, becoming kinetically sluggish.^1^ In a ground-breaking discovery in this field, Bourissou reported in 2014 that the oxidative addition of aryl iodides to gold­(I) was possible using chelation-assisted strategies with ligands that can bind as bidentate to the resulting gold­(III) complexes.^2,3^ The prevalent current strategies to achieve oxidative addition to gold­(I) complexes rely on the aforementioned chelation-assisted strategy or the use of bidentate ligands with small bite angles,^4^ strain release,^5^ as well as hemilabile ligands.^6^

However, there is seemingly a contradiction with the fact that, already in the early seventies, Schmidbaur,^7^ Kochi^8^ and Puddephatt^9^ reported the oxidative addition of alkyl halides to simple alkyl phosphine gold­(I) complexes such as [Ph_3_PAuMe], which by C–C reductive elimination yielded formally cross-coupled products (Scheme 1a).^10^ The proposed mechanism was that the gold­(III) complex emerging from oxidative addition undergoes ligand exchange with [LAuMe] to form [LAuMe_3_], which undergoes reductive elimination to form ethane. Reductive elimination from [Me_2_AuX] has also been observed.^8g^ These results had been, to a certain extent, reproduced by Levin and Toste in 2014 in the context of a cross-coupling of allyl bromides with boronic acids (Scheme 1b).^11^ In that report, however, the best results were obtained with a bimetallic catalyst bearing a bis­(phosphino)­amine ligand that favored an oxidative addition to form binuclear Au­(II) intermediates. On the other hand, oxidative addition of trifluoromethyl halides to gold­(I) to form gold­(II) complexes takes place by a photoinitiated radical process that involves gold­(II) intermediates.^12,13^

**Scheme 1:**
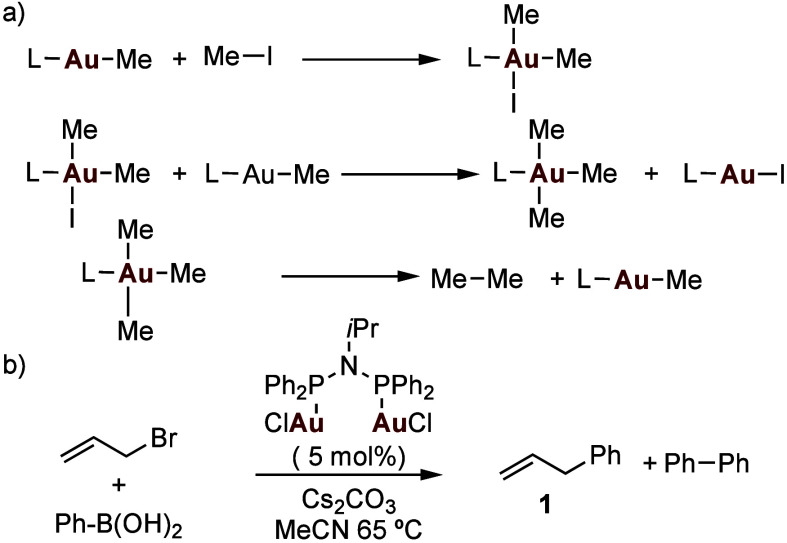
a) Oxidative Addition of MeI to Gold­(I) and Reductive Elimination.^8,9^ b) Gold­(I)-Catalyzed Coupling of Allyl Bromide with PhB­(OH)_2_ ^11^

Given this situation, we decided to study in detail the mechanisms behind the oxidative addition process when a simple alkyl phosphine is the ligand to gold­(I) complex. During this research, which makes heavy use of density functional theory (DFT), we have found some unexpected complications in terms of high sensitivity of the results with respect to the specific functional.

Based on the work of Levin and Toste,^11^ we expected that a coupling of cinnamyl bromide (**2**) with PhSnMe_3_ (**3**) would provide **4** (Scheme 2). Thus, after transmetalation of [LAuCl] with organostannane **3**, the resulting complex [LAuPh] (**5**)^14,15^ would undergo oxidative addition with **2** to form a gold­(III) intermediate **6**, which could undergo reductive elimination, closing a cross-coupling catalytic cycle to give **4** (Scheme 2a).^15^ In the event, reaction of nonvolatile cinnamyl bromide (**2**) (bp 212 °C) as the electrophile with organometallic nucleophile **3**, using 5 mol % of [Me_3_PAuCl], led to **4** in 33% yield after 48 h heating at 110 °C in deuterated toluene. A control experiment conducted in the absence of the gold­(I) complex led to 9% yield of **4** under the same conditions, meaning that a slow noncatalyzed background reaction might be taking place.

**Scheme 2:**
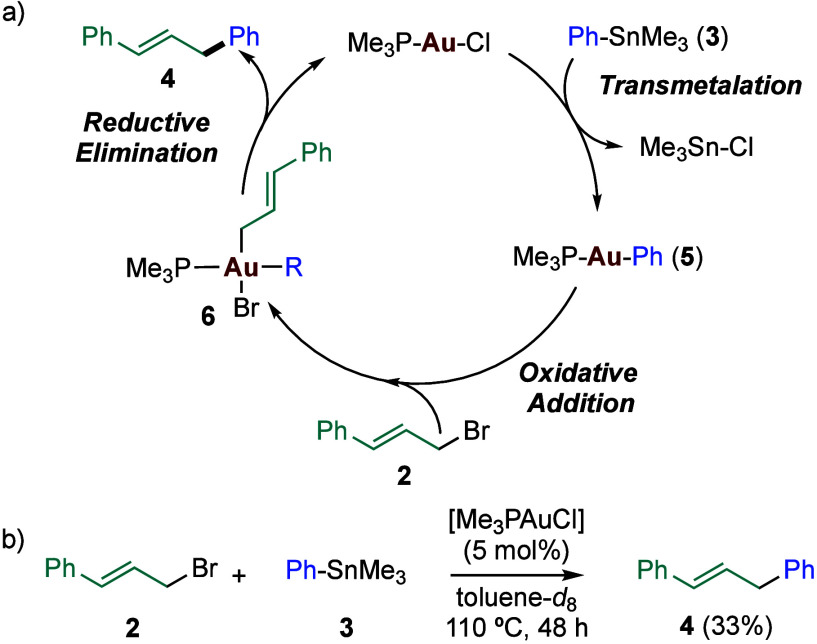
Simplified Cycle for Gold­(I)-Catalyzed Cross-Coupling of Cinnamyl Bromide with PhSnMe_3_

Based on these results, the system was explored computationally with the B3LYP-D3 ^16^ functional, and a plausible mechanism was found (Scheme 3). As the barriers seem quite high for the reported conditions, we decided to further explore the computational description. A batch of single point calculations with different functionals was performed on stationary points in Scheme 3, the results being reported in the . High variability of the data was observed, up to a difference of 20.2 kcal·mol^–1^.

**Scheme 3:**
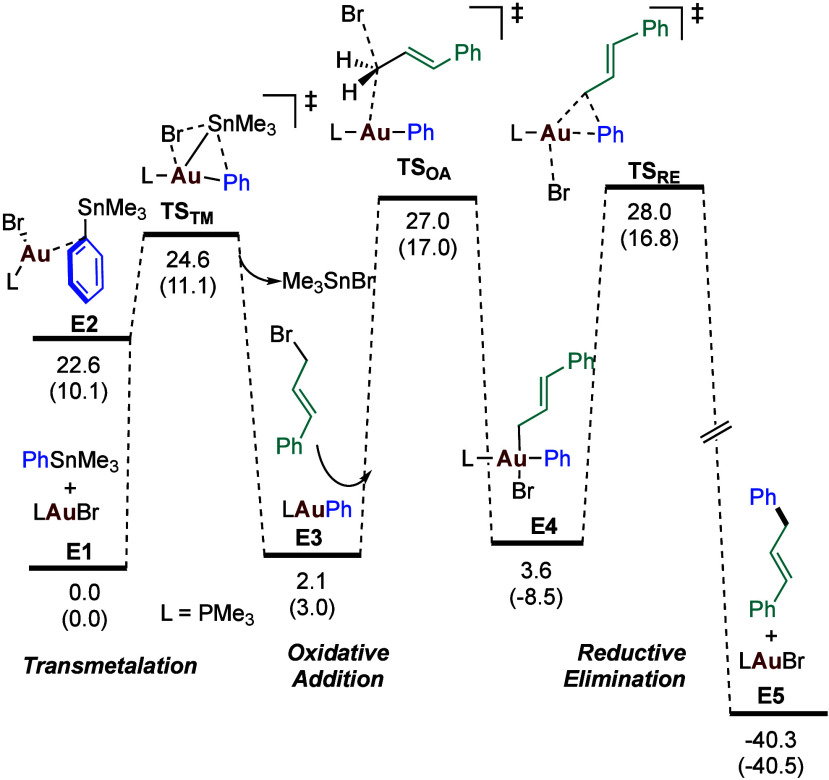
Mechanistic Pathway Submitted to DFT Benchmarking^a^

a B3LYP-D3. Free Energy above; potential energy in parentheses in kcal·mol^–1^. SMD (toluene).

Such a high range of computed energies was unexpected. There have certainly been reports of sensitivity of relative energies in transition metal chemistry associated with the amount of Hartree–Fock exchange in the functional,^17^ but this does not seem to be the key reason here. In the case of gold chemistry, the role of reactant distortion has been highlighted,^18^ but this does not seem to apply to this case either. So we carried out a further computational study with more functionals and including a more accurate wave function method such as CCSD­(T), see Table 1. For this second benchmarking exercise we moved from potential energies in solution to potential energies in vacuum to avoid possible distortions associated with the unbalanced consideration of solvation effects in the different methods.^19^ We are aware that it would be more desirable to make a comparison with reliable experimental kinetic data, but they are not available, and we think that the comparison between different computational methods is also informative.

**Table 1:** Benchmarking in Relative Potential Energy (kcal·mol^–1^) in Gas Phase Including DPLNO–CCSD­(T) as Reference

**Method**	**E2**	**TS**_**TM**_	**E3**	**TS**_**OA**_	**E4**	**TS**_**RE**_	**E5**
**B3LYP-D3**	6.8	7.9	2.1	17.0	–9.2	15.7	–39.9
**M06-L-D3** ^20^	7.9	7.7	3.9	17.8	–8.9	14.9	–40.6
**M06-D3**	6.0	9.4	4.5	23.8	–5.6	16.8	–40.1
**M062X-D3**	6.7	13.1	5.5	31.3	–3.0	19.9	–43.5
**M06-HF-D3** ^21^	5.2	9.1	4.0	32.7	–9.4	17.5	–43.5
**BMK-D3**	–1.0	2.0	1.4	21.1	–16.9	13.3	–42.0
**BP86-D3**	2.6	–0.2	0.2	9.4	–17.6	6.7	–38.1
**ωB97X-D**	8.0	12.1	4.0	26.9	–6.2	19.8	–41.3
**DPLNO–CCSD(T)**	8.2	11.5	2.1	32.9	–11.1	20.8	–38.0

The results obtained in the second benchmarking exercise in the gas phase follow the same trend as the ones in the first benchmark including solvation. Moreover, we can now evaluate the accuracy of the different functionals by comparing their performances to CCSD­(T). To quantify the comparison, we computed the root mean-square deviation (RMSD) of the differences in the predicted potential energies of each functional and DPLNO–CCSD­(T), see . The best functionals according to the lowest RMSD values are ωB97X-D and M06-HF-D3, while the worst functioning methods are BP86-D3 and BMK-D3.

Once we had established that the energetics for this system strongly depend on the specific functional, we decided to investigate how general this behavior is. With this goal, we examined another system involving gold catalysis. For an alternative gold system, we carried out a DFT benchmark (shown in the ) on the oxidative addition of PhI to [MeDalPhosAu­(SbF_6_)] to form a gold­(III) complex reported by Bourissou in 2017,^22^ which is the basis for the development of other catalytic systems based on gold­(I)/gold­(III).^23–25^ Again, the range of the computed potential energies for the oxidative addition step is up to 16 kcal·mol^–1^, which is a similar range as observed for the system outlined in Scheme 3 (up to 23.1 kcal·mol^–1^).

Our hypothesis for the origin of this high diversity in computed values for the energies is that it arises from something fundamental related to having different oxidation states^26^ gold­(I) and gold­(III). The first hint is that if we observe closely the results in Table 1, we see that with [Me_3_PAuCl] as reference, the energy differences of the gold­(III) species, transition states as well as intermediates, vary a lot, although the range of the relative energies of the gold­(I) species is not converging to more or less the same values. We admit here that a clarification of the microscopic origin of this difficulty of DFT in dealing with this problem goes beyond the scope of this manuscript.

A last question that we need to answer is whether the results of the functionals giving results closer to DLPNO–CCSD­(T) can reproduce the expected low free energy barrier in our systems of interest. This is not the case. The values in Table 1 may seem only slightly above expectations, with the highest barriers around 30 kcal·mol^–1^. However, these values correspond to potential energies, without entropic effects. When these are added to the CCSD­(T) potential energies, the relative Gibbs energy (ΔG^‡^) of the oxidative addition transition state using CCSD­(T) becomes 44.0 kcal·mol^–1^ in the gas phase. The values in solution, more difficult to estimate, lie in the same range. We also computed the original Kochi system,^8^ which covers the reaction of MeI with [Me_3_PAuCl]. The corresponding CCSD­(T) gas phase value for the TS was also calculated (see ), finding a free energy of 36.3 kcal·mol^**‑**1^, which is too high for the reaction to take place at 25 °C. Therefore, these reactions should occur by a different mechanism. Remarkably, the corresponding DLPNO–CCSD­(T) free energy barrier for the system developed by Bourissou is 17.7 kcal·mol^–1^ thus matching the reported experimental results obtained.

In conclusion, cross-coupling can take place between cinnamyl bromide (**2**) and PhSnMe_3_ in the presence of a simple gold­(I) catalyst with monophosphine ligands. The process, following the proposal by Kochi in the 1970s, can take place in both catalytic and stoichiometric conditions. Our attempts to characterize computationally the mechanism of the process have led to the identification of a complex problem with the DFT description of these systems. There is a high discrepancy, up to 23.1 kcal·mol^–1^, in the computed energies with widely used functionals in computational homogeneous catalysis. This divergence is most probably associated with the gold­(I)/gold­(III) conversion, as confirmed by calculations on another related system. A benchmark of the DFT results vs a DLPNO–CCSD­(T)/def2-TZVP calculation indicated that the best results are obtained with ωB97XD and M06-HF-D3. This work suggests caution about reaching mechanistic conclusions in catalytic reactions that involve gold­(I)/gold­(III) when using only one functional.

## Supplementary Material





## Data Availability

Computational data carried out for this study is available in the ioChem-BD repository and can be openly accessed at https://doi.org/10.19061/iochem-bd-1-382.

## Notes

Computational methods; experimental methods; additional calculations; additional experiments and total energies (PDF)
Cartesian coordinates of the calculated structures (XYZ)

## References

[1] Livendahl M., Goehry C., Maseras F., Echavarren A. M. (2014). Rationale
for the Sluggish Oxidative Addition of Aryl Halides to Au­(i). Chem. Commun..

[2] Guenther J., Mallet-Ladeira S., Estevez L., Miqueu K., Amgoune A., Bourissou D. (2014). Activation of Aryl Halides at Gold­(I):
Practical Synthesis
of (P,C) Cyclometalated Gold­(III) Complexes. J. Am. Chem. Soc..

[3] Joost M., Zeineddine A., Estevez L., Mallet-Ladeira S., Miqueu K., Amgoune A., Bourissou D. (2014). Facile Oxidative
Addition of Aryl Iodides to Gold­(I) by Ligand Design: Bending Turns
on Reactivity. J. Am. Chem. Soc..

[4] Joost M., Estévez L., Miqueu K., Amgoune A., Bourissou D. (2015). Oxidative
Addition of Carbon-Carbon Bonds to Gold. Angew.
Chem., Int. Ed. Engl..

[5] Wu C.-Y., Horibe T., Jacobsen C. B., Toste F. D. (2015). Stable Gold­(III)
Catalysts by Oxidative Addition of a Carbon-Carbon Bond. Nature.

[6] Chu J., Munz D., Jazzar R., Melaimi M., Bertrand G. (2016). Synthesis of Hemilabile Cyclic (Alkyl)­(Amino)­Carbenes
(CAACs) and Applications in Organometallic Chemistry. J. Am. Chem. Soc..

[7] Shiotani A., Schmidbaur H. (1972). Organogold-Chemie IX. Versuchezur Oxydative Addition
an Organogold-Komplexe. J. Organomet. Chem..

[8] Tamaki A., Kochi J. K. (1972). Catalytic Mechanism
InvolvingOxidative
Addition in the Coupling of Alkylgold­(I) with Alkyl Halides. J. Organomet. Chem..

[9] Johnson A., Puddephatt R. J. (1973). Oxidative Addition Reactions of Methylgold
(I) Compounds. Inorg. Nucl. Chem. Lett..

[10] Rocchigiani L., Bochmann M. (2021). Recent Advances in Gold­(III) Chemistry:
Structure,
Bonding, Reactivity, and Role in Homogeneous Catalysis. Chem, Rev..

[11] Levin M. D., Toste F. D. (2014). Gold-Catalyzed Allylation of Aryl
Boronic Acids: Accessing
Cross-Coupling Reactivity with Gold. Angew.
Chem., Int. Ed..

[12] Johnson A., Puddephatt R. J. (1976). Reactions of Trifluoromethyl Iodide with Methylgold­(I)
Complexes. Preparation of Trifluoromethyl-gold­(I) and -gold­(III) Complexes. J. Chem. Soc., Dalton Trans..

[13] Winston M. S., Wolf W. J., Toste F. D. (2014). Photoinitiated Oxidative Addition
of CF_3_I to Gold­(I) and Facile Aryl-CF_3_ Reductive
Elimination. J. Am. Chem. Soc..

[14] Carrasco D., García-Melchor M., Casares J. A., Espinet P. (2016). Dramatic mechanistic
switch in Sn/Au^I^ group exchanges: transmetalation vs. oxidative
addition. Chem. Commun..

[15] García-Melchor M., Braga A. A. C., Lledós A., Ujaque G., Maseras F. (2013). Computational
Perspective on Pd-Catalyzed C–C Cross-Coupling Reaction Mechanisms. Acc. Chem. Res..

[16] Becke A. D. (1993). Density-functional
Thermochemistry. III. The Role of Exact Exchange. J. Chem. Phys..

[17] Steinmetz M., Grimme S. (2013). Benchmark Study of the Performance of Density Functional
Theory for Bond Activations with (Ni,Pd)-Based Transition-Metal Catalysts. ChemistryOpen..

[18] Fernández I., Wolters L. P., Bickelhaupt F. M. (2014). Controlling the Oxidative Addition
of Aryl Halides to Au­(I). J. Comput. Chem..

[19] Pérez-Soto R., Besora M., Maseras F. (2020). The Challenge
of Reproducing with
Calculations Raw Experimental Kinetic Data for an Organic Reaction. Org. Lett..

[20] Zhao Y., Truhlar D. G. (2006). A New Local Density Functional for
Main-Group Thermochemistry,
Transition Metal Bonding, Thermochemical Kinetics, and Noncovalent
Interactions. J. Chem. Phys..

[21] Zhao Y., Truhlar D. G. (2006). Density Functional
for Spectroscopy: No Long-Range
Self-Interaction Error, Good Performance for Rydberg and Charge-Transfer
States, and Better Performance on Average than B3LYP for Ground States. J. Phys. Chem. A.

[22] Zeineddine A., Estévez L., Mallet-Ladeira S., Miqueu K., Amgoune A., Bourissou D. (2017). Rational Development of Catalytic Au­(I)/Au­(III) Arylation
Involving Mild Oxidative Addition of Aryl Halides. Nat. Commun..

[23] Rigoulet M., Thillaye du Boullay O., Amgoune A., Bourissou D. (2020). Gold­(I)/Gold­(III) Catalysis That
Merges Oxidative Addition and π-Alkene Activation. Angew. Chem., Int. Ed..

[24] Akram M. O., Das A., Chakrabarty I., Patil N. T. (2019). Ligand-Enabled Gold-Catalyzed C­(Sp2)–N Cross-Coupling
Reactions of Aryl Iodides with Amines. Org.
Lett..

[25] Zhang S., Wang C., Ye X., Shi X. (2020). Intermolecular Alkene
Difunctionalization via Gold-Catalyzed Oxyarylation. Angew. Chem., Int. Ed..

[26] Ducéré J.-M., Goursot A., Berthomieu D. (2005). Comparative Density Functional Theory Study of the
Binding of Ligands to Cu ^+^ and Cu ^2+^ :
Influence of the Coordination and Oxidation State. J. Phys. Chem. A.

