# Supplementary material for: Flash Communication: Challenging Computational Description of Gold(I)/Gold(III) Catalytic Cycles
Source: Organometallics. 2025 Jun 4;44(12):1241–5. doi: 10.1021/acs.organomet.5c00081 (PMC12188566; doi:10.1021/acs.organomet.5c00081)
Supplement: Supplementary file 1 [file om5c00081_si_001.pdf]

Supporting Information

Flash Communication:
Challenging Computational Description of Gold(I)/Gold(III) Catalytic Cycles

Isabel Arranz,[a,b] Feliu Maseras,[a,b]* and Antonio M. Echavarren [a,b]*

[a] Institute of Chemical Research of Catalonia (ICIQ-CERCA), The Barcelona Institute of Science and Technology, Av. Països Catalans 16, 43007 Tarragona, Spain.

[b] Departament de Química Analítica i Química Orgànica, Universitat Rovira i Virgili, C/Marcel·lí Domingo s/n, 43007 Tarragona, Spain.

Email:
aechavarren@iciq.es
fmaseras@iciq.es

Contents

1. DFT Calculations
    1.1. Computational Methods
    1.2. Computational Results
        1.2.1. First benchmarking
        1.2.2. Additional data on the second benchmarking
        1.2.3. Bourissou reported system
        1.2.4. Kochi's system
        1.2.5. Comparative of Free Energies calculated at DPLNO-CCSD(T) level
2. Experimental
    2.1. Experimental Methods
    2.2. General procedure for gold catalyzed allylation with cinnamyl bromide
    2.3. Catalyzed *vs* uncatalyzed reaction
3. Computed Structures and Energies

1. **DFT Calculations**

1.1. Computational Methods

Unless otherwise stated, all calculations were carried out using the Gaussian09 package.[1] All the reported energies are in kcal·mol^{-1}. Free energies quoted were calculated at default temperature 298.15 K and include a +1.89 kcal·mol^{-1} correction to reflect the 1M rather than ideal gas reference state.[2] Geometries were optimized to stationary points using the B3LYP[3] functional with Grimme's D3 dispersion correction[4] and the basis sets used were 6-31G(d,p)[5] for all elements except for Au, I, Br and Sn, where the SDD[6] basis set and ECP was employed. Single point calculations were performed with the cc-pVTZ[7] basis set for light atoms and cc-pVTZ-PP[8] basis set and ECP for Au, I, Br and Sn; using B3LYP-D3. Solvent effects were introduced with implicit model SMD[9] for toluene and iodomethane, using the same solvent in both optimization and single point calculations. All stationary points were confirmed through the absence of imaginary vibrations, and transition states were validated using IRC[10] calculations using LQA algorithm. A specific set of calculations was carried out a more accurate wavefunction method such as CCSD(T),[11] Additional single-point energy calculations were performed in gas phase on the previously optimized geometries using ORCA 4.0 software[12] at the DPLNO-CCSD(T) level with a larger def2-TZVP basis set (with ECP for internal electrons) for all atoms.

---

1 Gaussian 09, Revision B.1, Frisch, M. J., Trucks, G. W., Schlegel, H. B., Scuseria, G. E., Robb, M. A., Cheeseman, J. R., Scalmani, G., Barone, V., Mennucci, B., Petersson, G. A., Nakatsuji, H., Caricato, M., Li, X., Hratchian, H. P., Izmaylov, A. F., Bloino, J., Zheng, G., Sonnenberg, J. L., Hada, M., Ehara, M., Toyota, K., Fukuda, R., Hasegawa, J., Ishida, M., Nakajima, T., Honda, Y., Kitao, O., Nakai, H., Vreven, T., Montgomery, J. A., Peralta, Jr. J. E., Ogliaro, F., Bearpark, M., Heyd, J. J., Brothers, E., Kudin, K. N., Staroverov, V. N., Kobayashi, R., Normand, J., Raghavachari, K., Rendell, A., Burant, J. C., Iyengar, S. S., Tomasi, J., Cossi, M., Rega, N., Millam, J. M., Klene, M., Knox, J. E., Cross, J. B., Bakken, V., Adamo, C., Jaramillo, J., Gomperts, R., Stratmann, R. E., Yazyev, O., Austin, A. J., Cammi, R., Pomelli, C., Ochterski, J. W., Martin, R. L., Morokuma, K., Zakrzewski, V. G., Voth, G. A., Salvador, P., Dannenberg, J. J., Dapprich, S., Daniels, A. D., Farkas, Ö., Foresman, J. B., Ortiz, J. V., Cioslowski, J., Fox, D. J. Gaussian, Inc., Wallingford CT **2009**.

2 Luchini, G.; Alegre-Requena, J. V.; Funes-Ardoiz, I.; Paton, R. S. GoodVibes: Automated Thermochemistry for Heterogeneous Computational Chemistry Data, F1000Res **2020**, *9*, 291.

3 Density-functional Thermochemistry. III. The Role of Exact Exchange, *J. Chem. Phys.* **1993**, *98*, 5648–5652.

4 Grimme, S. Density Functional Theory with London Dispersion Corrections, *WIREs Computational Molecular Science* **2011**, *1*, 211–228.

5 Hehre, W. J.; Ditchfield, R.; Pople, J. A. Self—Consistent Molecular Orbital Methods. XII. Further Extensions of Gaussian—Type Basis Sets for Use in Molecular Orbital Studies of Organic Molecules, *J. Chem. Phys* **1972**, *56*, 2257–2261.

6 Andrae, D.; Häußermann, U.; Dolg, M.; Stoll, H.; Preuß, H. Energy-Adjustedab Initio Pseudopotentials for the Second and Third Row Transition Elements, *Theoret. Chim. Acta* **1990**, *77*, 123–141.

7 Kendall, R. A.; Dunning, T. H., Jr.; Harrison, R. J. Electron Affinities of the First-row Atoms Revisited. Systematic Basis Sets and Wave Functions, *J. Chem. Phys.* **1992**, *96*, 6796–6806.

8 Peterson, K. A.; Figgen, D.; Dolg, M.; Stoll, H. Energy-Consistent Relativistic Pseudopotentials and Correlation Consistent Basis Sets for the 4d Elements Y–Pd, *J. Chem. Phys.* **2007**, *126*, 124101.

9 Marenich, A. V.; Cramer, C. J.; Truhlar, D. G. Universal Solvation Model Based on Solute Electron Density and on a Continuum Model of the Solvent Defined by the Bulk Dielectric Constant and Atomic Surface Tensions, *J. Phys. Chem.* **2009**, *113*, 6378–6396.

10 Gonzalez, Carlos.; Schlegel, H. Bernhard. Reaction Path Following in Mass-Weighted Internal Coordinates, *J. Phys. Chem*. **1990**, *94*, 5523–5527.

11 Goerigk, L.; Grimme, S. A Thorough Benchmark of Density Functional Methods for General Main Group Thermochemistry, Kinetics, and Noncovalent Interactions, *Phys. Chem. Chem. Phys.* **2011**, *13*, 6670.

12 Neese, F. Software Update: The ORCA Program System, Version 4.0, *WIREs Comput Mol Sci* **2018**, *8*.

1.2. Computational Results

1.2.1 First benchmarking

The system in Scheme 3 of the main text was explored computationally with the B3LYP-D3. The first step is the transmetalation through a concerted transition state (**TS_{TM}**) with $\Delta G^{\ddagger}$ = 24.6 kcal·mol^{-1} to give phenyl–Au–PMe_3 (**E3**) as the species from which the oxidative addition of cinnamyl bromide (**2**) takes place (**TS_{OA}**, $\Delta G^{\ddagger}$ = 27.0 kcal·mol^{-1}). Finally reductive elimination (**TS_{RE}**, $\Delta G^{\ddagger}$ = 28.0 kcal·mol^{-1}) from the oxidative addition complex **E4** to yield the coupled product. These barriers seemed quite high for the experimental conditions, so we decided to further explore the computational description.

A batch of single point calculations was performed on stationary points in the free energy profile of Scheme 3. A first benchmarking was performed with different functionals, including pure (BP86[13]) and hybrid ones (BMK,[14] M06,[15] M062X, ωB97XD[16]). The basis set used was cc-pVTZ+cc-pVTZ-pp. The results of this initial benchmarking, based on potential energies (Table S1), are represented graphically in Scheme S1. A high diversity of the data was observed, up to a variation of 20.2 kcal·mol^{-1} (maximum value of 30.4 and minimum 10.2) on the relative energy of the transition state for the oxidative addition **TS_{OA}**.

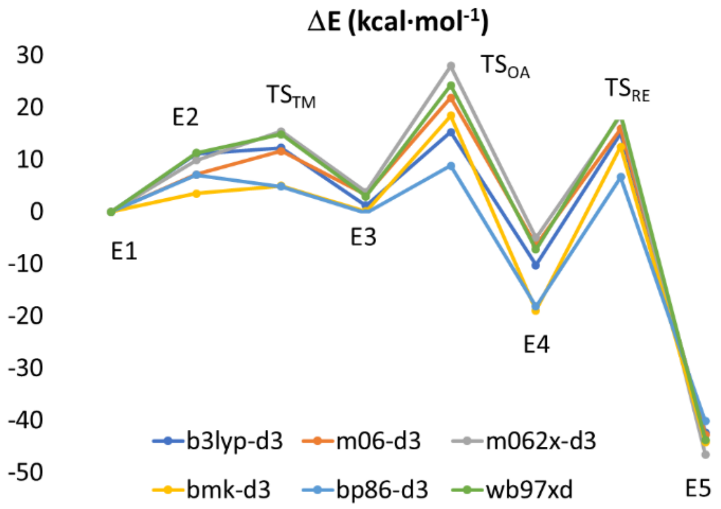

Scheme S1. Graphical Representation of Potential Energies (kcal·mol^{-1}) in the Benchmarking of the studied coupling.

13 Becke, A. D. Density-Functional Exchange-Energy Approximation with Correct Asymptotic Behavior, *Phys. Rev. A* **1988**, *38*, 3098–3100.

14 Boese, A. D.; Martin, J. M. L. Development of Density Functionals for Thermochemical Kinetics, *The Journal of Chemical Physics* **2004**, *121*, 3405–3416.

15 Zhao, Y.; Truhlar, D. G. The M06 Suite of Density Functionals for Main Group Thermochemistry, Thermochemical Kinetics, Noncovalent Interactions, Excited States, and Transition Elements: Two New Functionals and Systematic Testing of Four M06-Class Functionals and 12 Other Functionals, *Theor Chem Account* **2008**, *120*, 215–241.

16 Chai, J.-D.; Head-Gordon, M. Long-Range Corrected Hybrid Density Functionals with Damped Atom–Atom Dispersion Corrections, *Phys. Chem. Chem. Phys.* **2008**, *10*, 6615.

Table S1. Benchmarking of DFT methods in relative potential energy (kcal·mol^{-1}) for the gold-catalyzed cross-coupling reaction of cinnamyl bromide (**2**) with **3**. SMD (toluene).

Method	E2	TS_{TM}	E3	TS_{OA}	E4	TS_{RE}	E5
**B3LYP-D3**	10.1	11.1	3.0	17.0	-8.5	16.8	-40.5
**M06-D3**	9.0	12.9	5.3	23.9	-4.5	17.9	-40.7
**M062X-D3**	10.2	16.4	6.2	30.4	-2.5	20.8	-44.1
**BMK-D3**	2.0	4.7	1.7	20.1	-17.3	14.1	-42.5
**BP86-D3**	5.9	3.0	1.1	10.2	-16.8	8.0	-38.8
**ωB97XD**	11.2	15.3	4.7	26.0	-5.5	20.6	-41.9

1.2.2 Additional Data on the Second Benchmarking

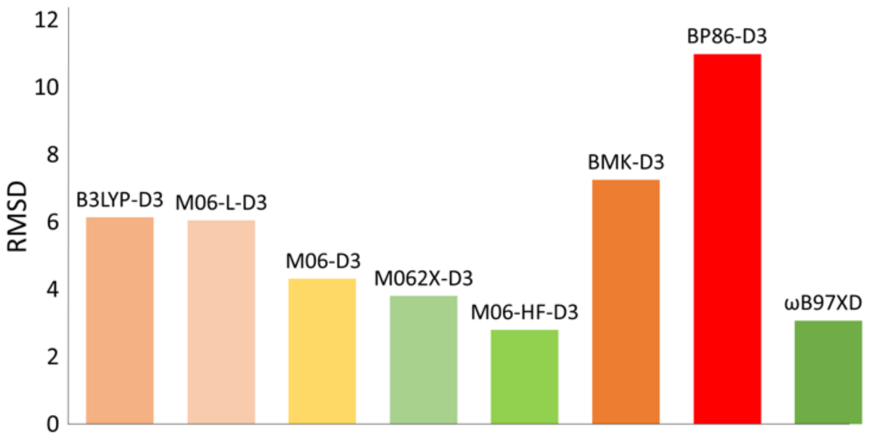

Scheme S2. Root-Mean-Square Deviation (RMSD) of the Differences Between the Predicted Potential Energies with Functional-D3/def2-TZVP and DLPNO-CCSD(T), in Gas Phase

1.2.3 Bourissou reported system

Once established that the energetics for this system highly depend on the specific functional, we decided to investigate how general is this behaviour. With this goal, we examined another system involving gold catalysis. For an alternative gold system, we carried out a DFT benchmarking on the oxidative addition of PhI to [MeDalPhosAu(SbF_6)] (**7**) to form gold(III) complex **9** reported by Bourissou in 2017 (Scheme S3),[17] which is the basis for the development of other catalytic

17 Zeineddine, A.; Estévez, L.; Mallet-Ladeira, S.; Miqueu, K.; Amgoune, A.; Bourissou, D. Rational Development of Catalytic Au(I)/Au(III) Arylation Involving Mild Oxidative Addition of Aryl Halides, *Nat Commun* **2017**, *8*, 565.

systems based on gold(I)/gold(III).[18,19,20] This reaction takes place stepwise via intermediate **8**, which undergoes oxidative addition through **TS$_{8\text{-}9}$**. Again, the dispersion of the computed potential energies for the oxidative addition step is up to 16 kcal·mol^{-1}, which is similar dispersion compared to the system outlined in Scheme 3 (up to 23.1 kcal·mol^{-1}).

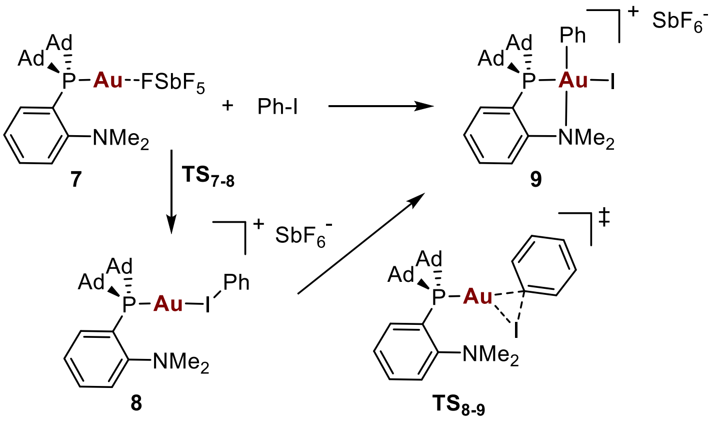

Scheme S3. Oxidative Addition of PhI to [MeDalPhosAuCl] (**7**) to Form Au(III) Complex.[17]

Table S2. Benchmarking in relative potential energy (kcal·mol^{-1}) in gas phase for the oxidative addition of PhI to **7** to form **9** including DPLNO-CCSD(T) as reference. Basis set is def2-TZVP.

Method	TS$_{7\text{-}8}$	8	TS$_{8\text{-}9}$	9
**B3LYP-D3**	-8.8	-15.7	-0.5	-17.6
**M06-D3**	-12.6	-18.3	-2.3	-11.4
**M062X-D3**	-8.7	-12.5	7.3	-5.8
**BMK-D3**	-15.1	-21.8	-6.0	-26.5
**BP86-D3**	-11.5	-19.1	-9.1	-27.0
**ωB97X-D**	-8.3	-15.0	2	-14.0
**DPLNO-CCSD(T)**	-4.7	-12.3	4.9	-18.5

18 (a) Rigoulet, M.; Thillaye du Boullay, O.; Amgoune, A.; Bourissou, D. Gold(I)/Gold(III) Catalysis That Merges Oxidative Addition and π-Alkene Activation. *Angew. Chem. Int. Ed.* **2020**, *59*, 16625–16630. (b) Rodriguez, J.; Adet, N.; Saffon-Merceron, N.; Bourissou, D. Au(I)/Au(III)-Catalyzed C–N Coupling. *Chem. Comm.* **2020**, *56*, 94–97. (c) Rigoulet, M.; Miqueu, K.; Bourissou, D. Mechanistic Insights about the Ligand-Enabled Oxy-Arylation/Vinylation of Alkenes via Au(I)/Au(III) Catalysis. *Chem. Eur. J.* **2022**, *28*, e202202110.
19 (a) Akram, M. O.; Das, A.; Chakrabarty, I.; Patil, N. T. Ligand-Enabled Gold-Catalyzed C(Sp2)–N Cross-Coupling Reactions of Aryl Iodides with Amines. *Org. Lett.* **2019**, *21*, 8101–8105. (b) Chintawar, C. C.; Yadav, A. K.; Patil, N. T. Gold-Catalyzed 1,2-Diarylation of Alkenes. *Angew. Chem. Int. Ed.* **2020**, *59*, 11808–1181. (c) Tathe, A. G.; Chintawar, C. C.; Bhoyare, V. W.; Patil, N. T. Ligand-Enabled Gold-Catalyzed 1,2-Heteroarylation of Alkenes. *Chem. Commun.* **2020**, *56*, 9304–9307. (d) Tathe, A. G.; Urvashi; Yadav, A. K.; Chintawar, C. C.; Patil, N. T. Gold-Catalyzed 1,2-Aminoarylation of Alkenes with External Amines. *ACS Catal.* **2021**, *11*, 4576–4582. (e) Tathe, A. G.; Patil, N. T. Ligand-Enabled Gold-Catalyzed C(Sp^2)–S Cross-Coupling Reactions. *Org. Lett.* **2022**, *24*, 4459–4463. (f) Kumar, A.; Patil, N. T. Ligand-Enabled Sustainable Gold Catalysis. *ACS Sust. Chem. Eng.* **2022**, *10*, 6900–69.
20 Zhang, S.; Wang, C.; Ye, X.; Shi, X. Intermolecular Alkene Difunctionalization via Gold-Catalyzed Oxyarylation. *Angew. Chem. Int. Ed.* **2020**, *59*, 20470–20474.

1.2.4 Kochi's system

The oxidative addition of iodomethane to $H_3C–AuPR_3$ **A1** was studied (Scheme S4) and after evaluating other mechanisms such as a concerted one, the most favorable was found to occur via an S_N2 reaction (**TSA2**).

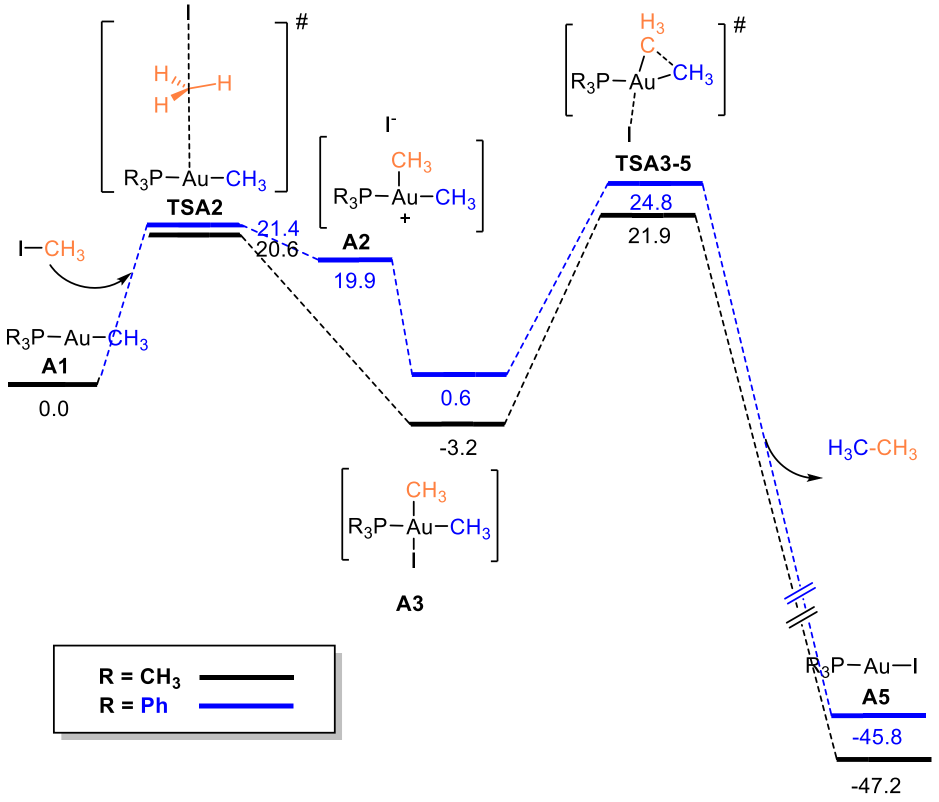

Scheme S4. Oxidative Addition and Reductive Elimination in the Coupling of alkylgold(I) **A1** with methyl iodide. Free Energies in kcal·mol^{-1}. SMD (iodomethane)

1.2.5 Comparative of Free Energies calculated at DPLNO-CCSD(T) level

Having analysed the unexpected effect on the potential energies the different systems, we wanted to take into consideration the relative Gibbs free energy ($\Delta G^{\#}$) of the oxidative addition transition states using CCSD(T) (Table S3).

Table S3. Free energies in kcal·mol^{-1} using CCSD(T) method in gas phase for the oxidative addition transition states of the studied systems

	$\Delta G^{\#}$ (kcal·mol^{-1})		
	Our system	Kochi	Bourissou
**CCSD(T)**	44.4	36.3	17.7

2. Experimental

2.1. Experimental Methods

Most reagents and solvents were purchased from commercial sources and used without further purification. Cinnamyl bromide was purified until 97% purity as a white solid by distillation (Kügelrohr: 122 ºC, 2 mbar) from commercially available reagent, before using in reactions. The dry solvents used were passed through an activated alumina column on a PureSolvTM Solvent Purification System (SPS, Innovative Technologies, Inc., MA), or purchased from ACROS Organics as commercially available anhydrous solvents. The reaction monitoring was followed by NMR analysis, TLC (thin layer chromatography), UHPLC–MS (Agilent Technologies 1290 Infinity II, LC/MS with single–quad detector InfinityLab (APCI ionization source)) or by GC–MS. Thin layer chromatography was carried out using TLC aluminum sheets coated with 0.2 mm of silica gel (Merck Gf234), using UV light as the visualizing agent and an acidic solution of vanillin in ethanol or alternatively a basic solution of KMnO4 in water as stain, followed in both cases by heat. The chromatographic purifications were carried out using flash grade silica gel (SDS Chromatogel 60 ACC, 40–60 μm) as the stationary phase. Preparative thin layer chromatography was performed on TLC plates (Analtec Silica Gel GF UV254, 20×20 cm, 1000 μm or 2000 μm). NMR spectra were recorded at 298 K on Bruker Avance Ultrashield NMR spectrometers (300 MHz, 400 MHz, 500 MHz, and 500 MHz with CryoProbe). Chemical shifts (δ) are reported in parts per million (ppm) and referenced to residual solvent (For ^{1}H NMR: $CDCl_3$ at 7.26 ppm, CD_2Cl_2 at 5.31 ppm, C_6D_6 at 7.16 ppm, for ^{13}C{^{1}H} NMR: $CDCl_3$ at 77.16 ppm, CD_2Cl_2 at 54.00 ppm, C_6D_6 at 128.06 ppm). The following abbreviations were used to explain multiplicities: s = singlet, d = doublet, t = triplet, q = quartet, p = pentet, m = multiplet, br s = broad singlet. Coupling constants (J) are reported in Hertz (Hz). Mass spectra were recorded on a Waters LCT Premier Spectrometer (ESI and APCI) or on an Autoflex Broker Daltonics (MALDI and LDI).

2.2. General procedure for gold catalyzed allylation with cinnamyl bromide.

In a MW vial equipped with a stirring bar, $ClAuP(CH_3)_3$ was weighted and added as a solid. $PhSn(CH_3)_3$ was added using a Hamilton syringe and 3 cycles of vacuum/Ar were performed. Then, cinnamyl bromide was added as a solution in toluene-d_8 and heated up to 110 ºC for 48 h. The reaction mixture was allowed to cool down to 25 ºC, the internal standard was added and submitted to ^{1}H and ^{31}P NMR spectroscopy.

The suitable conditions to be able to calculate the NMR yield properly were achieved when deuterated toluene was used in order to avoid evaporation of starting material, product or even internal standard.

2.3. Catalyzed vs uncatalyzed reaction

An experiment was designed to see the difference between the gold(I)-catalyzed reaction and the uncatalyzed reaction. Two separate reactions were set under the same conditions and monitored by ^{1}H NMR, one adding 5 mol% of $ClAuPEt_3$ (Figure S1, blue dots) and a second one lacking the gold(I) complex (Figure S1, orange dots). As observed, when gold(I) is present, the formation of product is relatively fast, achieving 30% yield after 16 h, while a rather slow conversion to product **3** takes place reaching 8% after 3 days at 110 ºC. At this point after 48 h, 5 mol% of $ClAuPEt_3$ was added to the same reaction vessel and a clear increase in the rate formation of product **3** is seen.

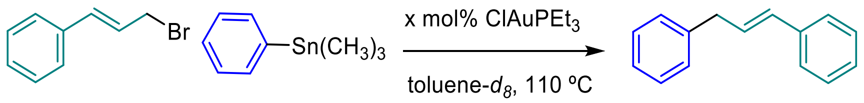

1 **2** **3**

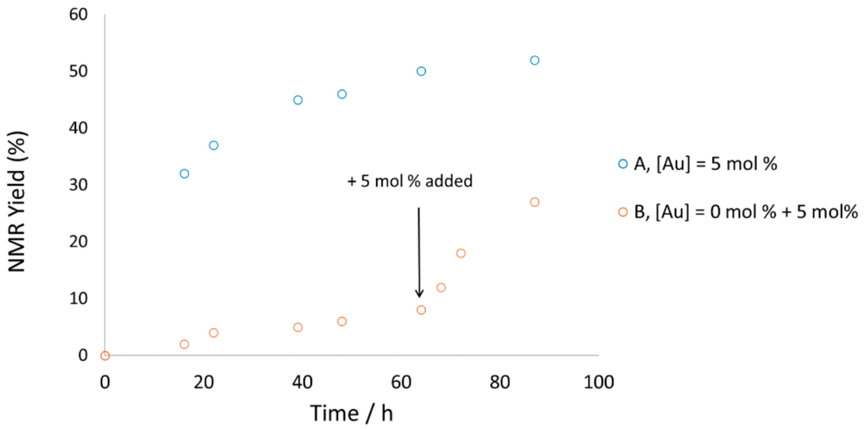

Figure S1. Plot representing the concentration of product **3** along reaction time. NMR yield calculated with respect to ethyl benzoate as internal standard

Reaction conditions: [SM] = 100 mM, [$ClAuPEt_3$] = 0/0/5 mM. Three MW vials equipped with magnetic stirrer containing substrate (cinnamyl bromide **1**), ethyl benzoate as internal standard, trimethyl(phenyl)stannane **2** and gold complex $ClAuPEt_3$ in deuterated toluene (3.6 mL) were prepared. Precisely, the following reagents were added in sequence into the different MW vials:

1) Substrate, internal standard and trimethyl(phenyl)stannane stock solution (3.6 mL), previously prepared by adding deuterated toluene (13.8 mL) to the substrate (272.4 mg, 1.38 mmol), organostannane (275 mL, 1.52 mmol) and internal standard (207.6 mg, 1.38 mmol).

2) $ClAuPEt_3$ (0/0/6.3 mg), obtaining a clear yellowish solution.

After preparing the solution, the reaction vessel was sealed and heated up to 110 ºC. The aliquotes for the kinetic monitoring were taken from the previously cooled to 25 ºC MW vials (leaving one of the non-containing gold vials untouched during 64 h) and directly transferred to NMR tubes for measuring the corresponding ^{1}H NMR spectra (8 scans). After 64 h of reaction, in the untouched vial without gold, 6.3 mg of $ClAuP(CH_3)_3$ were added and the reaction was monitored until no more evolution to product **3**.

3. Computed Structures and Energies

Table S4. Oxidative addition and reductive elimination in the coupling of (R_3P)$AuCH_3$ with methyl iodide reported by Kochi. B3LYP-D3/6- 31G(d,p)+SDD(Au, Br, Sn); SMD (iodomethane). HLT: B3LYP-D3/cc-pVTZ+cc-pVTZ-PP (Au, Br, Sn); SMD (iodomethane)

	R	E / Hartree	G / Hartree	E_{HLT} / Hartree
CH_3I		-51.3348	-51.3208	-335.7390
CH_3CH_3		-79.8408	-79.7861	-79.8680
**A1**	CH_3	-636.8716	-636.7554	-636.9601
**TSA2**		-688.1953	-688.0504	-972.6811
**A3**		-688.2324	-688.0842	-972.7223
**TSA3-5**		-688.1933	-688.0482	-972.6791
**A5**		-608.4478	-608.3686	-892.9100
**A1**	Ph	-1,212.1138	-1,211.8530	-1,212.3884
**TSA2**		-1,263.4388	-1,263.1476	-1,548.1097
**A2**		-1,263.4414	-1,263.1505	-1,548.1119
**A3**		-1,263.4722	-1,263.1768	-1,548.1470
**TSA3-5**		-1,263.4343	-1,263.1421	-1,548.1053
**A5**		-1,183.6891	-1,183.4641	-1,468.3372

Table S5. Oxidative addition, reductive elimination and transmetalation processes, including intermediates and transition states. B3LYP-D3/6- 31G(d,p)+SDD(Au, Br, Sn); SMD (toluene). HLT: B3LYP-D3/cc-pVTZ+cc-pVTZ-PP (Au, Br, Sn); SMD (toluene)

	E / Hartree	G / Hartree	E_{HLT} / Hartree
**2**	-354.7975	-354.6373	-565.9115
**E1**	-610.4051	-610.3247	-1014.0210
**E2**	-965.2002	-964.9397	-1579.9165
**TS_{TM}**	-965.1933	-964.9312	-1579.9149
**$SnBr(CH_3)_3$**	-136.5766	-136.5019	-751.1570
**E3**	-828.6189	-828.4543	-828.7708
**1**	-361.7805	-361.6616	-765.4384
**TS_{OA}**	-1190.3905	-1190.0898	-1594.1868
**E4**	-1190.4205	-1190.1165	-1594.2275
**TS_{RE}**	-1190.3865	-1190.0839	-1594.1872
**3**	-580.0647	-579.8598	-580.2576

Table S6. Benchmark of geometries. Functional-D3/cc-pVTZ+cc-pVTZ-PP(Au, Br, Sn); SMD (toluene). Potential energies in Hartrees.

	**2**	**E1**	**E2**	**TS_{TM}**	**$SnBr(CH_3)_3$**	**1**
**B3LYP**	-565.9115	-1014.0210	-1579.9165	-1579.9149	-751.1570	-765.4384
**M06**	-565.6029	-1013.8585	-1579.4470	-1579.4408	-751.0723	-765.1547
**M062X**	-565.5859	-1013.8461	-1579.4157	-1579.4058	-751.0508	-765.3718
**BMK**	-564.7384	-1011.7452	-1576.4804	-1576.4762	-748.7555	-763.7669
**BP86**	-565.9383	-1014.2298	-1580.1588	-1580.1633	-751.2946	-765.5277
**ωB97XD**	-565.7634	-1013.9683	-1579.7138	-1579.7072	-751.1031	-765.2924

	**E3**	**TS_{OA}**	**E4**	**TS_{RE}**	**3**
**B3LYP**	-828.7708	-1594.1868	-1594.2275	-1594.1872	-580.2576
**M06**	-828.3806	-1593.5057	-1593.5509	-1593.5152	-579.7502
**M062X**	-828.3713	-1593.7045	-1593.7570	-1593.7199	-579.9771
**BMK**	-827.7254	-1591.4631	-1591.5226	-1591.4725	-579.8176
**BP86**	-828.8718	-1594.3850	-1594.4280	-1594.3884	-580.2332
**ωB97XD**	-828.6210	-1593.8795	-1593.9297	-1593.8881	-580.0195

Table S7. Second benchmark of geometries. Functional-D3/def2-TZVP; gas phase. Potential energies in Hartrees.

	**2**	**E1**	**E2**	**TS_{TM}**
**B3LYP**	-565.9089	-3171.2769	-3737.1749	-3737.1732
**M06**	-565.9169	-3171.1292	-3737.0335	-3737.0338
**M06**	-565.6058	-3170.9725	-3736.5687	-3736.5634
**M062X**	-565.5783	-3170.9966	-3736.5642	-3736.5540
**M06-HF**	-565.5130	-3171.1208	-3736.6255	-3736.6193
**BMK**	-564.7399	-3169.0753	-3733.8168	-3733.8120
**BP86**	-565.9368	-3171.6312	-3737.5639	-3737.5683
**ωB97XD**	-565.7616	-3171.2552	-3737.0041	-3736.9976
**DPLNO-CCSD(T)**	-564.3500	-3168.4125	-3732.7494	-3732.7441

	$SnBr(CH_3)_3$	1	E3
**B3LYP**	-2908.4136	-2922.6921	-828.7688
**M06**	-2908.3433	-2922.4941	-828.6965
**M06**	-2908.1859	-2922.2674	-828.3853
**M062X**	-2908.2044	-2922.5219	-828.3617
**M06-HF**	-2908.2715	-2922.7871	-828.3559
**BMK**	-2906.0872	-2921.0968	-827.7257
**BP86**	-2908.6951	-2922.9255	-828.8725
**ωB97XD**	-2908.3898	-2922.5766	-828.6207
**DPLNO-CCSD(T)**	-2905.9115	-2920.3815	-826.8476

	TS_{OA}	E4	TS_{RE}	3
**B3LYP**	-3751.4372	-3751.4789	-3751.4393	-580.2510
**M06**	-3751.1684	-3751.2111	-3751.1731	-580.1323
**M06**	-3750.6219	-3750.6688	-3750.6330	-579.7512
**M062X**	-3750.8426	-3750.8972	-3750.8607	-579.9651
**M06-HF**	-3751.0973	-3751.1644	-3751.1216	-580.0979
**BMK**	-3748.7911	-3748.8516	-3748.8035	-579.8163
**BP86**	-3751.7834	-3751.8264	-3751.7876	-580.2279
**ωB97XD**	-3751.1608	-3751.2136	-3751.1721	-580.0143
**DPLNO-CCSD(T)**	-3747.1801	-3747.2501	-3747.1993	-578.8805
